# TET-catalyzed 5-hydroxymethylcytosine regulates gene expression in differentiating colonocytes and colon cancer

**DOI:** 10.1038/srep17568

**Published:** 2015-12-03

**Authors:** Christopher G. Chapman, Christopher J. Mariani, Feng Wu, Katherine Meckel, Fatma Butun, Alice Chuang, Jozef Madzo, Marc B. Bissonnette, John H. Kwon, Lucy A. Godley

**Affiliations:** 1 Section of Gastroenterology, Department of Medicine, The University of Chicago, Chicago, IL 60637, USA; 2 Section of Hematology/Oncology, Department of Medicine, The University of Chicago, Chicago, IL 60637, USA; 3 Committee on Molecular Pathogenesis and Molecular Medicine, The University of Chicago, Chicago, IL 60637, USA; 4 Committee on Cancer Biology, The University of Chicago, Chicago, IL 60637, USA

## Abstract

The formation of differentiated cell types from pluripotent progenitors involves epigenetic regulation of gene expression. DNA hydroxymethylation results from the enzymatic oxidation of 5-methylcytosine (5-mC) to 5-hydroxymethylcytosine (5-hmC) by the ten-eleven translocation (TET) 5-mC dioxygenase enzymes. Previous work has mapped changes in 5-mC during differentiation of intestinal stem cells. However, whether or not 5-hmC regulates colonocyte differentiation is unknown. Here we show that 5-hmC regulates gene expression during colonocyte differentiation and controls gene expression in human colon cancers. Genome-wide profiling of 5-hmC during *in vitro* colonic differentiation demonstrated that 5-hmC is gained at highly expressed and induced genes and is associated with intestinal transcription factor binding sites, including those for HNF4A and CDX2. *TET1* induction occurred during differentiation, and *TET1* knockdown altered gene expression and inhibited barrier formation of colonocytes. We find that the 5-hmC distribution in primary human colonocytes parallels the distribution found in differentiated cells *in vitro*, and that gene-specific 5-hmC changes in human colon cancers are directly correlated with changes in gene expression. Our results support a model in which 5-hmC regulates differentiation of adult human intestine and 5-hmC alterations contribute to the disrupted gene expression in colon cancer.

Differentiation of the intestinal epithelium along the spatially distinct crypt-luminal axis requires gene expression changes mediated in part by epigenetic pathways^1^. Modified cytosine bases include 5-methylcytosine (5-mC), 5-hydroxymethylcytosine (5-hmC), 5-formylcytosine (5-fC), and 5-carboxylcytosine (5-caC) and are one mechanism of epigenetic regulation of gene expression^2^,^3^,^4^,^5^. Changes to 5-mC have previously been implicated in regulation of intestinal differentiation. Specifically, differentially methylated regions have been identified between the stem and differentiated cells of the mouse small intestine. Co-localization of these regions with lineage-specific transcription factor binding sites and enhancer regions suggests that these 5-mC changes play an important role in intestinal differentiation^6^,^7^. Similar observations in hematopoietic and skin cell differentiation suggest that this is a common feature of lineage commitment^8^,^9^.

5-mC is converted to 5-hmC by the ten-eleven-translocation 5-methylcytosine dioxygenase (TET) enzymes^2^,^3^. Although accumulation of 5-mC at gene promoters and enhancers has been associated with gene repression^10^, 5-hmC has distinct biological functions. 5-hmC has been associated with active genes, has been proposed to play a role in numerous demethylation pathways, and may serve as a bona fide epigenetic mark with a distinct set of binding proteins^11^. Recently, mapping of 5-hmC in differentiation of erythroid cells and studies of *Tet2* knockout mice have demonstrated that TET activity is critical for normal hematopoietic differentiation^12^,^13^. However, the importance of TET activity in the differentiation of other cell types remains unclear.

To determine if 5-hmC has a functional role in regulating colonocyte differentiation, we mapped 5-hmC changes during cell-cell adhesion-initiated differentiation of T84 colon adenocarcinoma cells since a similar system had previously been used to map chromatin regulatory regions of small intestinal differentiation^14^. When seeded at low density, these contact-naïve cells proliferate to form a confluent monolayer consisting of polarized cells with high transepithelial electrical resistance and morphological, structural, functional, and transcriptional features of colonocytes *in vivo*^15^,^16^. We extend our studies to human samples by comparing our *in vitro* 5-hmC maps to the 5-hmC profile of primary human colonocytes. Finally, since developmental pathways are frequently dysregulated in cancer^17^, we define regions losing and gaining 5-hmC in human colon cancers, and correlate these alterations with changes in gene expression.

## Results

### 5-hmC is increased during T84 cell differentiation and is associated with epithelial pathways and transcription factor binding sites

**Figure 1:**
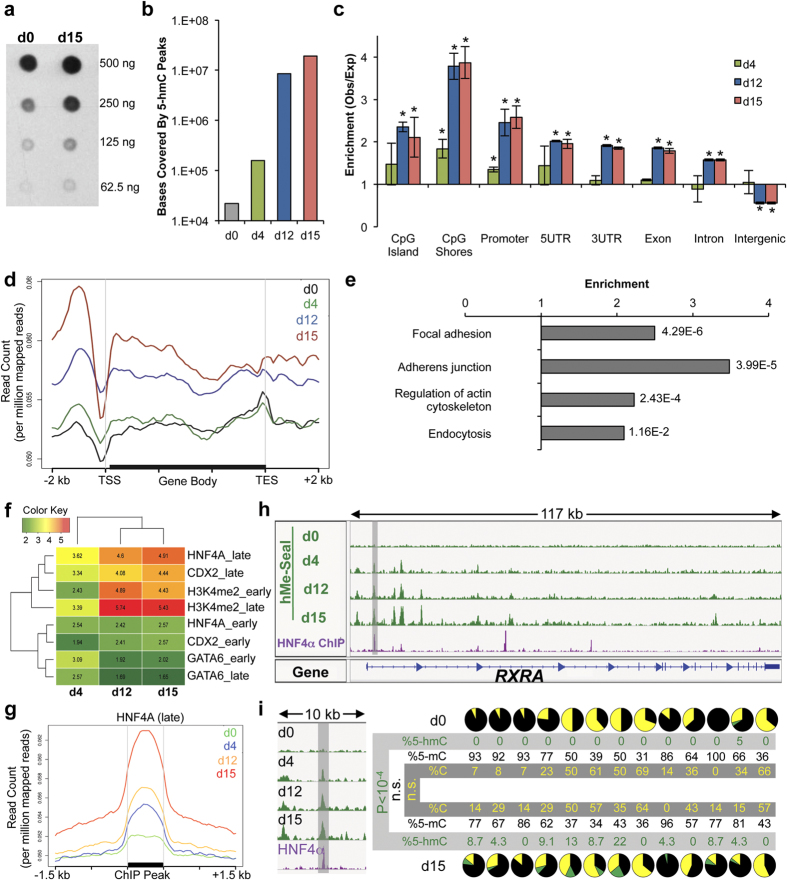
5-hmC is gained during differentiation at epithelial associated genes and transcription factor binding sites. (**a**) Dot blot analysis of 5-hmC quantity at days 0 and 15 of differentiation. Dot blot performed by biotinylation of 5-hmC followed by probing with Avidin-HRP. Dot blot representative of three replicates. (**b**) The number of bases covered by 5-hmC peaks at each day of differentiation. Data represent averages from two replicates. (**c**) Enrichment of 5-hmC at different genomic annotations. For each time point, enrichment is determined by calculating the observed overlap of hMe-Seal sequencing tags with each genomic element and then dividing this number by the expected value of overlap assuming a random distribution of 5-hmC. See methods for details. Error bars represent standard deviations of two replicates. *p < 0.001 in random permutation tests. (**d**) The 5-hmC profile over an average RefSeq gene at each time point. Data represent one replicate, for correlation between replicates see Fig. S1. (**e**) KEGG pathway analysis of genes with 5-hmC peaks at day 15. Values to the right of bar graphs represent FDR-corrected p-values. (**f**) Enrichment of 5-hmC at early and late binding sites of GATA6, CDX2, and HNF4A as well as positions marked by H3K4me2. hMe-Seal data acquired in T84 cells. Positions of GATA6, CDX2, HNF4A, and H3K4me2 were previously determined by ChIP-seq in proliferating and differentiated Caco2 cells (GSE23436)^14^. Enrichment values calculated by random permutation tests. (**g**) hMe-Seal sequencing profile around late HNF4A binding sites. hMe-Seal performed in T84 cells. HNF4A binding sites in differentiated Caco2 cells were previously published (GSE23436)^14^. (**h**) hMe-Seal and HNF4A ChIP-seq data at *RXRA*. hMe-Seal data acquired using T84 cells. HNF4A ChIP-seq data are from differentiated Caco2 cells (GSE23436)^14^. (**i**) TAB-seq data from the region shaded in (h) (hg19; chr9:137,220,139-137,220,610). From left to right, each pie chart represents a single CpG. Pie charts represent percent 5-mC (black), 5-hmC (green), and cytosine (gold). Percent 5-mC, 5-hmC, and cytosine were calculated following sequencing of at least 16 clones generated by PCR amplification of bisulfite or Tet-oxidized and bisulfite treated DNA. P-values calculated by chi-squared tests. See also Fig. S1 and Table S1.

T84 cells were seeded at low density and transepithelial electrical resistance was used to monitor T84 cell monolayer formation as cells differentiated (Fig. S1a). Total 5-hmC levels increased in differentiated cells (day 15) relative to proliferating cells (day 0) by dot blot assay (Fig. 1a). We used the hMe-Seal method to isolate and sequence 5-hmC-enriched DNA from cells at days 0, 4, 12, and 15 to determine how 5-hmC distribution changed during differentiation (Fig. S1b–e)^18^. Consistent with our dot blot results, we found that 5-hmC covered an increasing amount of the genome and that hMe-Seal peaks became more intense as differentiation progressed (Fig. 1b and Fig. S1f–j). We found no enrichment of 5-hmC at various genomic elements (CpG islands, CpG shores, promoters, 5′ UTRs, exons, introns, 3′ UTRs, and intergenic regions) at day 0, but significant enrichment at CpG shores and promoter regions by day 4. By day 12 and day 15, a strong 5-hmC signature was observed, with significant enrichment for 5-hmC over CpG islands, CpG shores, promoters, and gene bodies (Fig. 1c). We observed a relative preference for 5-hmC at CpG shores relative to CpG islands (Fig. S1k). To visualize 5-hmC changes over genes, we plotted the 5-hmC profile of an average gene at each time point. This demonstrated that 5-hmC was gained over promoters and gene bodies (Fig. 1d). KEGG pathway analysis demonstrated that 5-hmC peaks were enriched at genes involved in epithelial barrier function, including focal adhesion, adherens junctions, regulation of actin cytoskeleton, and endocytosis (Fig. 1e).

5-hmC frequently colocalizes with transcription factor binding sites^12^,^19^. Therefore, we analyzed genomic sequences covered by 5-hmC and found that they were predicted to bind the HNF4A, RXRA, and CDX2 transcription factors, which are known to regulate intestinal development (Table S1)^20^. We validated this result against ENCODE HNF4A ChIP-seq data acquired from HepG2 cells (Fig. S1l)^21^.

Previous work mapped early and late binding sites of HNF4A, CDX2, and GATA6 as well as the active enhancer mark H3K4me2 during differentiation of the Caco2 colon cancer cell line^14^. We calculated enrichment of 5-hmC at these regions and found that 5-hmC becomes especially enriched at the late binding sites of HNF4A and CDX2. We also observed enrichment of 5-hmC at enhancer regions (Figs 1f,g and S1m). GATA6 binding sites, served as a negative control and showed only weak overlap with 5-hmC. Furthermore, we examined HNF4A binding sites by Tet-assisted bisulfite sequencing (TAB-seq)^22^, which allows for quantification of cytosine, 5-mC, and 5-hmC at single base resolution. We performed TAB-seq at the HNF4A binding sites of *RXRA*, *GNA12*, *VAV2* and at auto-regulatory sites of *HNF4A* (Figs 1h,i and S1n–t). In addition to gain of 5-hmC, we observed demethylation at the binding sites of *VAV2* and *GNA12.* HNF4A binding sites have been associated with regions losing methylation in intestinal differentiation^7^. Since 5-hmC is implicated in demethylation pathways^11^, our data indicate that TET activity at these positions is a mechanism for this observation.

### 5-hmC is associated with highly expressed and induced genes

**Figure 2:**
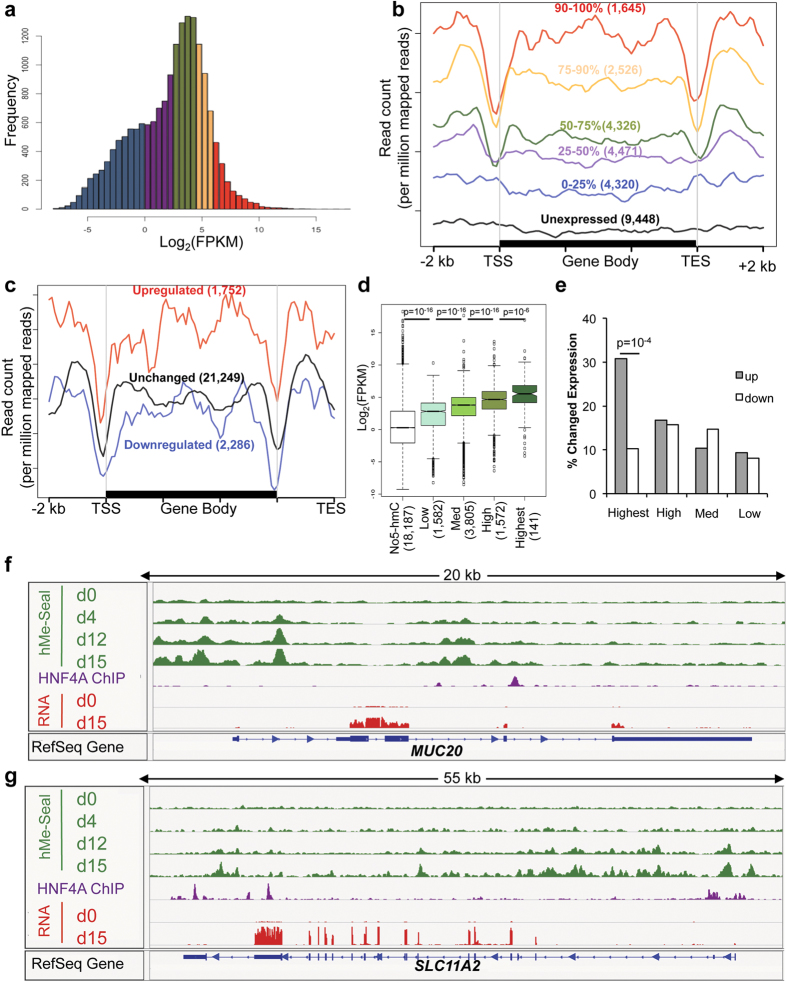
5-hmC is associated with highly expressed and induced genes. (**a**,**b**) Genes were separated by expression level at day 15 (**a**) and their 5-hmC profiles were plotted (**b**). The number of genes included in each subgroup is indicated in parentheses. (**c**) 5-hmC profile of upregulated, downregulated, and unchanged genes. The number of genes included in each subgroup is indicated in parentheses. (**d,e**) Genes were separated by 5-hmC density (see Fig. S2b) and expression level of these genes at day 15 (**d**) and percent of these genes up- or down-regulated over the time course (**e**) are plotted. The number of genes included in each subgroup is indicated in parentheses. P-value calculated by chi-squared test. (**f,g**) Examples of genes that gain expression and 5-hmC. HNF4A ChIP-seq data represent a previously published data set obtained from differentiated Caco2 cells (GSE23436)^14^. Genome-wide sequencing data presented in Fig. 2 are representative of one of two biological replicates. For correlation between replicates, see Figure S1.

The increase of 5-hmC in differentiation, association of 5-hmC with important epithelial pathways, and colocalization of 5-hmC with lineage-specific intestinal transcription factor binding sites implicated 5-hmC gains as a regulator of colonocyte differentiation. To identify the relationship between 5-hmC and gene expression, we used RNA-seq to quantify transcript levels at each time point of differentiation. KEGG pathway analysis showed that genes associated with MAPK signaling were induced, and numerous metabolic and disease-associated pathways were repressed (Fig. S2a). We separated genes by expression level at day 15 (Fig. 2a), and found a positive correlation between 5-hmC and expression (Fig. 2b). To test the relationship between gene induction over the time course and 5-hmC levels, we identified genes with changed expression and plotted 5-hmC levels. We found that up-regulated genes had higher 5-hmC levels than genes that were down-regulated or had unchanged expression over the time course (Fig. 2c). To confirm these correlations, we performed the inverse analyses and examined expression as a function of 5-hmC. Highly hydroxymethylated genes were more highly expressed (Figs 2d and S2b), and genes with the highest levels of 5-hmC were more likely to be induced (Fig. 2C), and had a greater median fold change than genes with lower 5-hmC levels (Fig. S2c). Examples of genes that gain both 5-hmC and expression include *MUC20*, *SLC11A2*, *SLC26A2*, *SLC6A8*, *FXYD1*, and *NDRG1* (Figs 2f,g and S2d–g). Association of 5-hmC with highly expressed and induced genes suggested that 5-hmC had an important role in regulation of gene expression during differentiation.

### TET1 regulates gene expression in differentiating colonocytes

**Figure 3:**
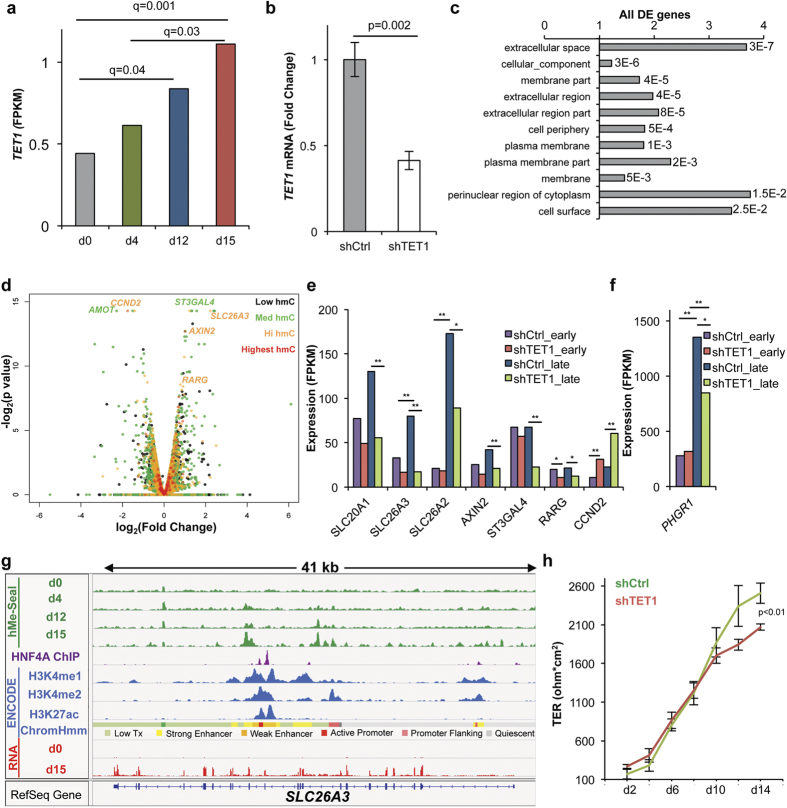
TET1 regulates gene expression in differentiating T84 cells. (**a**) *TET1* expression over the differentiation time course measured by RNA-seq in fragments per kilobase of transcript per million mapped reads (FPKM). Q-values represent FDR-adjusted p-values outputted by Cuffdiff. RNA-seq data from two biological replicates were used as input into the Cuffdiff program. (**b**) T84 cells stably expressing a *TET1* targeting shRNA were generated using lentivirus. *TET1* knockdown was quantified by qPCR (n = 3). Error bars represent standard deviations. P-value calculated by Student’s t-test. (**c**) GO cellular compartment analysis of all dysregulated genes in shTET1 relative to shCtrl cells at day 15. Values to the right of bar graphs represent FDR-corrected p-values. (**d**) Volcano plot analysis of differentially expressed genes between shTET1 and shCtrl cells at day 15. Analysis includes only genes with hydroxymethylation at day 15. Data represent analysis of RNA-seq data from two biological replicates. Fold change and p-values were outputted from Cuffdiff. (**e,f**) RNA-seq measured expression of selected genes with both hydroxymethylation and differential expression at day 15. Expression is measured as FPKM. Test statistics are FDR-corrected outputs from Cuffdiff analysis using two biological replicates, *q < 0.05, **q < 0.01. (**g**) Integration of 5-hMe-Seal data acquired in differentiating T84 cells with other epigenetic features at *SLC26A3.* Data for histone ChIP-seq and ChromHMM segmentation were obtained from ENCODE data sets from HepG2 cells (GSM798321, GSM733693, GSM33743). HNF4A ChIP-seq data represent a previously published dataset acquired using differentiated Caco2 cells differentiated (GSE23436)^14^. (**h**) TER measurements for T84 cells expressing a control or *TET1* targeting hairpin. Error bars represent standard deviations, p-value calculated by Student’s t-test (n = 3). See also Figure S3.

To test for functional significance of TET activity during differentiation, we performed *TET1* knockdown experiments since *TET1* was induced during differentiation (Fig. 3a, S3a,b) and since *TET1* has been implicated as a tumor suppressor and regulator of the WNT pathway in carcinomas, including colon cancer^23^. Differential expression analysis of cells expressing a control versus *TET1* targeting hairpin at day 15 (Fig. 3b) revealed that differentially regulated genes were enriched for genes coding for proteins targeted to the cell membrane and extracellular space (Fig. 3c). To identify potential direct *TET1* targets, we identified differentially expressed genes that were hydroxymethylated, including: *SLC26A3*, *SLC20A1*, *SLC26A2*, *AXIN2*, *ST3GAL4*, *RARG*, and *PHGR1* (Figs 3d–f, S3c,d). Given the exquisite sensitivity of *SLC26A3* to *TET1* knockdown, we integrated the 5-hmC profile of this gene with ENCODE data from HepG2 cells to infer the epigenetic context of 5-hmC at this locus. This analysis revealed that 5-hmC is gained at a region marked by H3K4me1, H3K4me2, and H3K27ac and is defined as an enhancer region by the ChromHMM model of chromatin segmentation (Fig. 3g)^24^. To determine if the gene expression changes observed on *TET1* knockdown had a functional consequence on colonocyte function, transepithelial electrical resistance (TER) measurements were performed on cells depleted of *TET1*. We found that *TET1* knock down attenuated TER at late time points (Fig. 3h), demonstrating that gene expression changes introduced on *TET1* knockdown compromised barrier formation *in vitro*.

### The 5-hmC distribution of primary human colonocytes is similar to that of differentiated T84 cells and is altered in human cancers in association with gene expression changes

**Figure 4:**
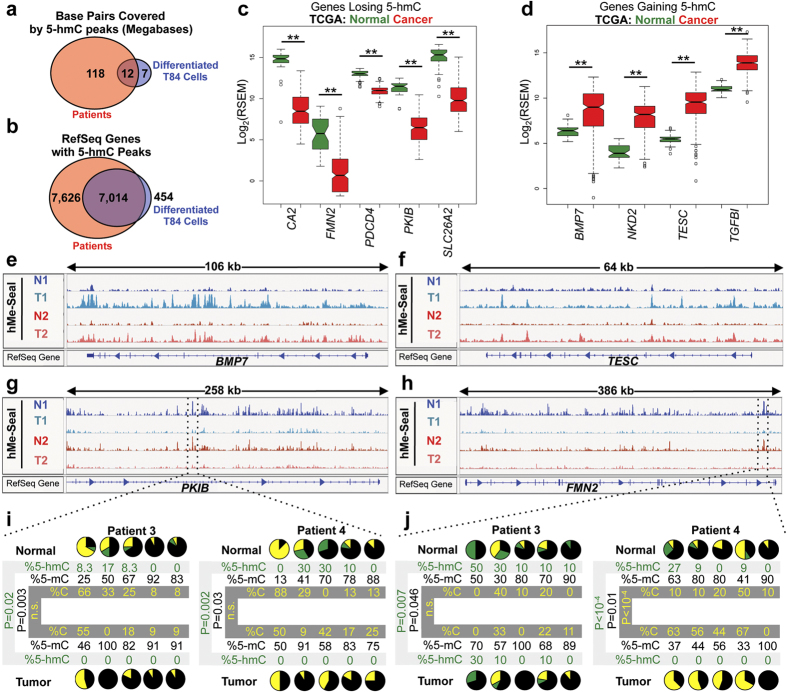
5-hmC regulates similar genes *in vivo* and is dysregulated in colon cancer. (**a**) Comparison of genomic regions covered by 5-hmC in differentiated colonocytes *in vitro* and primary human patient samples. Overlap is measured in number of bases with a called peak in both the *in vivo* and *in vitro* samples. Analysis was performed by first identifying regions with peaks in both normal patient samples and then comparing these positions to regions with peaks in both T84 cell replicates at day 15. (**b**) Comparison of genes with 5-hmC peaks in differentiated colonocytes *in vitro* and primary human patient samples. Genes with 5-hmC peaks were identified as any gene having a peak called by MACS1.4 in its gene body. Genes with peaks in both normal patient samples were identified and compared with genes with peaks in both T84 cell replicates. (**c,d**) Expression of selected genes losing and gaining 5-hmC in cancer. Data acquired from the TCGA. Units of expression are RNA-seq by expectation-maximization (RSEM). Test statistics were calculated by Wilcoxon rank-sum tests. **p < 0.001 following Bonferonni correction. **(e–h)** Examples of genes that lose and gain 5-hmC in paired tumor and adjacent normal samples. N1, N2 normal 1 and 2; T1, T2 tumor 1 and 2. (**I,j**) TAB-seq results of the region denoted in (g) and (h) (hg19; chr6:122,890,582-122,890,851 and chr1:240,622,715-240,622,914). From left to right, each pie chart represents a single CpG at day 0 or day 15. In pie charts, black represents 5-mC, green represents 5-hmC, and gold represents cytosine. Experiments performed using independent patient samples. P-values calculated by chi-squared tests. See also Figure S4.

To compare the 5-hmC profile observed *in vitro* with the distribution found *in vivo*, we performed hMe-Seal on isolated colonocyte fractions from two patients. We found that more regions of the genome were covered by 5-hmC peaks in primary human colonocytes than in differentiated T84 cells, consistent with a previous report that 5-hmC levels are lower in cell lines^25^. Despite the larger amount of 5-hmC found *in vivo*, sixty-five percent of regions covered by 5-hmC *in vitro* were also hydroxymethylated *in vivo* (Fig. 4a). Moreover, nearly all genes with a 5-hmC peak *in vitro* also had a 5-hmC peak *in vivo* (Fig. 4b). This analysis demonstrated that genes regulated by 5-hmC *in vitro* are also regulated by 5-hmC *in vivo*. Like our *in vitro* data, patient samples had high 5-hmC levels at HNF4A binding sites and at genes in similar KEGG pathways (Fig. S4a–e). These results show that while 5-hmC may regulate more genes *in vivo* the biological function is likely similar.

Altered regulation of developmental pathways such as the WNT, Hedgehog, Notch, and BMP pathways is recognized as a hallmark of cancer^17^. Since our results delineate a role for 5-hmC in regulating normal differentiation, we tested whether or not 5-hmC distribution is altered in primary human colon cancers by performing cell-type specific hMe-Seal on isolated colonocytes from matched colon cancer and adjacent normal samples to identify genes that lose or gain 5-hmC in cancer (Fig. S4f). Using The Cancer Genome Atlas (TCGA) dataset, we found that 163 of 222 genes losing 5-hmC in cancer also lost expression, and that 96 of 175 genes gaining 5-hmC in cancer gained expression (Fig. S4g). Examples of genes with hydroxymethylation and gene expression changes include: *CA2*, *FMN2*, *PDCD4*, *PKIB*, *SLC26A2*, *BMP7*, *NKD2*, *TESC*, and *TGFBI* (Figs 4c–h and S4h–l). Gene expression changes for these genes were further validated using qPCR (Fig. S4m). We further quantified changes in 5-hmC, 5-mC, and cytosine in additional independent patient samples at *PKIB* and *FMN2* using TAB-seq. Consistent loss of 5-hmC at these loci demonstrates that this is a common feature of colon cancers (Fig. 4i,j).

Given that we observed gene-specific changes in 5-hmC, we performed pathway analysis to determine if certain KEGG pathways were enriched for gaining or losing 5-hmC in cancer. Surprisingly, we could not identify pathways that were significantly enriched for genes gaining or losing 5-hmC in the colon cancer samples relative to adjacent normals. Since we had identified the focal adhesion, adherens junctions, regulation of actin cytoskeleton, and endocytosis pathways as being enriched for 5-hmC in differentiated T84 cells, we determined if these pathways had alterations to 5-hmC levels in cancer. Plotting 5-hmC levels over genes in these pathways revealed that there were no consistent changes of 5-hmC levels over these pathways (Fig. S5), demonstrating that while gene-specific changes may contribute to altered expression in cancer, consistent pathway-wide changes in 5-hmC could not be identified.

## Discussion

Our results identify a previously unappreciated role for 5-hmC in regulating epithelial differentiation. We observe accumulation of 5-hmC at intestine specific genes and transcription factor binding sites. Integration of hMe-Seal and RNA-seq data demonstrates that 5-hmC is particularly enriched at highly expressed and/or induced genes, and *TET1* knockdown shows functional significance of TET activity in colonocyte differentiation. Mapping of 5-hmC in primary colonocytes and cancers demonstrates that perturbations to TET activity are a mechanism of altered expression in cancer.

Previous studies have profiled cytosine modifications during differentiation of small intestinal epithelium using bisulfite-based techniques unable to distinguish 5-mC and 5-hmC^6^,^7^,^26^,^27^. These experiments revealed relatively few changes between the stem and differentiated populations. In contrast, our studies reveal that gains in 5-hmC during differentiation are relatively common. This suggests that transitions between 5-mC and 5-hmC, which would not be detected by bisulfite-based techniques, may be more frequent than transitions between modified (5-mC and 5-hmC) and unmodified cytosines.

Co-localization of 5-hmC with lineage-specific transcription factor binding sites suggests either that multiple transcription factors direct TET enzymes to target loci or that TET activity is required for subsequent transcription factor binding. Previous work has demonstrated that CDX2 functions as a pioneer transcription factor triggering chromatin remodeling^28^. Although we find greater enrichment of 5-hmC at HNF4A binding sites, these transcription factors frequently regulate the same regions^14^. Future work should explore the molecular interactions between these transcription factors and the TET enzymes, and determine how TET activity at these regions is coordinated with other aspects of chromatin remodeling. Our data at *SLC26A3* and regions marked by H3K4me2 suggest that TET activity is likely integrated with other chromatin modifying enzymes at enhancer regions. Future work should interrogate the role of each TET enzyme in regulating distinct chromosomal regions and gene sets during epithelial differentiation.

Previous reports have identified 5-hmC loss as a hallmark of cancer, including colon cancer^23^,^29^,^30^,^31^,^32^,^33^,^34^. However, many of these studies measured only global 5-hmC content without genome-wide mapping. Our studies reveal both locus-specific losses and gains of 5-hmC in cancer. These 5-hmC changes occur at genes coding for proteins involved in regulating intestinal development and cancer cell proliferation. For example, we demonstrate significant loss of 5-hmC at *FMN2*, a gene whose protein product promotes cell cycle arrest by inhibiting the degradation of p21^35^. 5-hmC gains occur at genes including *NKD2*, a gene coding for a regulator of Wnt and TGFa/EGFR signaling^36^,^37^,^38^, and *BMP7*, a member of the TGF-beta superfamily^39^. Gain of 5-hmC also occurs at *TGFBI*, a gene whose over-expression in colon cancer has been considered as a prognostic biomarker and target for therapy since TGFBI promotes metastasis^40^. Similarly, *TESC* codes for a member of the calcineurin homologous protein family that has been reported to activate NF-κB and possibly Akt signaling pathways to control the survival and proliferation of colorectal cancer cells^41^.

Correlation of 5-hmC alterations with tumor-associated changes in gene expression suggests that 5-hmC changes can contribute to both gene activation and repression in cancer. These epigenetic lesions are of particular interest since they are potentially reversible with epigenetic targeting agents, and future work should determine whether or not any of these locus-specific 5-hmC changes predict response to these drugs. Finally, by defining regions with altered 5-hmC in colon cancer, our work provides a starting point for identifying key genomic regions that could be epigenetically altered in other epithelial malignancies and advances our understanding of the molecular mechanisms underlying tumorigenesis.

## Methods

### T84 *in vitro* differentiation cell culture

T84 human colon carcinoma epithelial cells were grown in biologic triplicate on tissue culture-treated 6-well Transwell filter support units (0.4-μm pore size). The culture medium employed was a 1:1 mixture of Dulbecco’s modified Eagle’s medium (DMEM) and Ham’s F-12 nutrient mixture supplemented with heat-inactivated fetal bovine serum (FBS) and 100 U/mL penicillin/0.1 mg/mL streptomycin. The cells were grown in a humidified 5% CO_2_ atmosphere at 37**°**C. Culture media was replaced approximately every 48 hours. The monolayers were grown until achieving differentiation with transepithelial resistance (TER) measurements of >2,000 Ω ⋅ cm^2^.

### Transepithelial electrical resistance (TER) measurement

TER was measured with a MilliCell-ERS volt-ohmmeter (Millipore) and chopstick current electrodes approximately every 48 hours prior to culture medium exchange. All TER values were normalized by subtracting the baseline resistance measurement across the Transwell filter support unit in the absence of cells.

### Quantification of 5-hmC by dot blot

Dot blot assays were performed as previously described^19^. Briefly, genomic DNA was sonicated, 5-hmC was conjugated to UDP-6-N3-Glu^18^, and biotinylated by addition of 4.5 nm Click-IT Biotin DIBO Alkyne (Life Technologies). The biotin labeled DNA was purified and spotted on HyBond N + membrane (GE Healthcare Life Sciences). Membranes were probed with Avidin-HRP and dots were visualized with Western Lightning ECL reagent (PerkinElmer).

### 5-hydroxymethylcytosine selective chemical labeling (hMe-Seal)

hMe-Seal followed previously published protocols^18^,^19^. Briefly, 20 μg genomic DNA was sonicated to 250 base pairs (Covaris), conjugated to UDP-6-N3-Glu with β-GT, and purified with Micro Bio-Spin Columns (Bio-Rad). 5-hmC was subsequently biotinylated by addition of DMCO-S-S-PEG3-Biotin Conjugate (Click Chemistry Tools). Biotinylated DNA was isolated using streptavidin-coated magnetic beads (Invitrogen). After washing beads, labeled DNA was released with 50 mM DTT and purified sequentially with Micro Bio-Spin Columns (Bio-Rad) and MinElute reaction cleanup kits (Qiagen). The concentration of the isolated DNA was measured by Qubit (Qiagen).

### hMe-Seal read mapping and peak calling

Reads were mapped to the hg19 genome using the BWA- backtrack algorithm. Peak calling was performed using MACS 1.4.

### Enrichment calculations for 5-hmC at genomic elements

Enrichment for 5-hmC at genomic regions used previously described permutation analyses^19^.

Genomic coordinates of 5′ UTRs, 3′ UTRs, exons, introns, and CpG islands were obtained from the UCSC Table Browser^42^. Promoter regions were defined by downloading TSS sites from the UCSC Table Browser and using the bedtools suite to generate a bed file of coordinates 2 kb downstream to 500 bp upstream of TSSs. Genomic coordinates of CpG shores were deduced from the locations of CpG islands by using the bedtools suite to define regions 2 kb adjacent to each CpG island.

To calculate enrichment of 5-hmC over each annotation, each peak identified by MACS 1.4 was annotated with the number of tags mapping to that region (also outputted by MACS 1.4). The number of tags mapping to each peak was divided by the length of that peak to determine tags/bp over that peak. The peak bed file was then intersected against genomic annotations. For each intersection of a peak and a genomic annotation, the length of the intersection in bases was multiplied with the number of tags per base for that peak island in order to obtain the number of sequencing tags at each intersection:

(Length of the intersection between peak and genomic annotation in bps) × (sequencing tags per base at peak island) = sequencing tags over intersection

Summing these values for all intersections genome-wide gives the absolute number of sequencing tags over each annotation being analyzed. This value served as an “observed” value for enrichment calculations.

To calculate enrichment, the observed value was divided by an expected value. The expected value was determined by scrambling 5-hmC peaks across the genome using the bedtools shuffle command and repeating the calculation described above. Shuffling was performed 1,000 times for each enrichment analysis, and the average value obtained from these random permutations was used as the expected value in enrichment calculations.

For all enrichment calculations, each biological replicate was treated independently, and results averaged to give enrichment values at each time point and over each annotation.

### KEGG pathway analysis

A list of genes with 5-hmC peaks within 2 kb of their gene body was generated by first downloading the positions of all RefSeq genes from the UCSC table browser. The bedtools slop command was used to lengthen the positions of these genes by 2 kb. This file was then intersected with the output of MACS 1.4 peaks at day 15. The gene list outputted by this command was used in KEGG pathway analysis performed on the DAVID Bioinformatics Resources 6.7 website (https://david.ncifcrf.gov/).

### Genomic positions of *GATA6*, *HNF4A*, *CDX2*, and H3K4me2 in proliferating and differentiated cells

Positions of these transcription factor binding sites and histone modification have previously been determined in Caco2 cells^14^. The positions of these features were acquired from GEO (GSE23436). Peaks were converted from hg18 to hg19 coordinates using the UCSC liftover tool.

### Quantification of 5-hmC over genes

To quantify 5-hmC over gene bodies, a gtf file was created with positions of all RefSeq genes. For genes with a MACS 1.4 called peak over their gene body, Ht-seq count was used to sum hMe-Seal reads over these genes. To control for gene length, the number of 5-hmC sequencing tags mapping to each gene was then divided by the length of the gene to give 5-hmC density over each gene. Plotting the distribution of gene density yielded the histogram in Fig. S2b. The histogram demonstrated a normal distribution with a right tail. Therefore, genes were binned by 0-25, 25-75, 75-98, and 98–100 percentile.

### Plotting hMe-Seal data around genomic elements

To visualize 5-hMe-Seal sequencing data around a given genomic feature (e.g., gene bodies, CpG islands, and transcription factor binding sites), the ngs-plot software was used^43^. Positions of CpG islands were obtained from the UCSC table browser. Positions of transcription factor binding sites were acquired from previously published datasets as described above. Sam files aligned by bwa were used as input.

### Tet-assisted bisulfite sequencing (TAB-seq)

TAB-Seq was performed using the Wisegene 5-hmC TAB-Seq kit according to the manufacturer’s protocol. Bisulfite treatment was performed using the MethylCode Bisulfite Conversion Kit (Life Technologies, MECOV-50). PCR reactions used ZymoTaq (Zymo Research). PCR products were cloned using the NEB PCR cloning kit (NEB E1202S).

### RNA-seq read mapping and expression analysis

RNA-seq analysis followed a published protocol^44^. Reads were mapped to the hg19 genome using Bowtie2 and TopHat2. Gene expression quantification was performed using Cufflinks to calculate fragments per kilobase of transcript per million mapped reads (FPKM), and Cuffdiff to perform differential expression analysis. All gene analyses used a GTF file obtained from Ensembl.

### Generation of T84 cells stably expressing shRNAs

Production of lentiviral particles and subsequent infection of target cells followed the pLKO.1 protocol found at http://www.addgene.org/tools/protocols/plko/. Infection was performed with a final polybrene concentration of 8 μg/mL. Cells expressing the desired shRNAs were selected using puromycin (8 μg/mL, Santa Cruz). pLKO.1 plasmids coding for *TET1* targeting hairpins were previously described^45^.

### Analysis of the cancer genome atlas (TCGA) data

Level 3 TCGA data were downloaded from the TCGA data matrix. Gene level RSEM data, calculated by the TCGA, were used to measure expression. Heat maps were generated by the UCSC cancer browser^46^. Wilcoxon-Rank sum tests were used to test for differential expression on TCGA data. Test statistics were Bonferonni corrected.

### Preparation of isolated colonocytes from surgically resected mucosa

We adapted a method to isolate a nearly pure population of colonocytes^47^. Colon crypts from surgically resected mucosa stripped from muscle of four patients with moderately differentiated sporadic colon cancer and adjacent normal colon were isolated by EDTA/EGTA at 4 °C. Crypts were released by gentle shaking and separated from stromal cells by centrifugation at 40 g for 2 min. Isolated crypts stained for positive (CK20) and negative (vimentin) markers.

## Additional Information

**Accession Numbers**: Next generation sequencing datasets are available at http://www.ncbi.nlm.nih.gov/geo: GSE55391.

**How to cite this article**: Chapman, C. G. *et al.* TET-catalyzed 5-hydroxymethylcytosine regulates gene expression in differentiating colonocytes and colon cancer. *Sci. Rep.* **5**, 17568; doi: 10.1038/srep17568 (2015).

## Supplementary Material

Supplementary Data
