# Corrigendum: TET-catalyzed 5-hydroxymethylcytosine regulates gene expression in differentiating colonocytes and colon cancer

**DOI:** 10.1038/srep24963

**Published:** 2016-04-28

**Authors:** Christopher G. Chapman, Christopher J. Mariani, Feng Wu, Katherine Meckel, Fatma Butun, Alice Chuang, Jozef Madzo, Marc B. Bissonnette, John H. Kwon, Lucy A. Godley

Scientific Reports 5: Article number: 17568;10.1038/srep17568 published online: 12032015; updated: 04282016

The original version of this Article contained a typographical error in the spelling of the author Marc B. Bissonnette, which was incorrectly given as Marc B. Bissonette. This has now been corrected in the PDF and HTML versions of the Article.

