# Supplementary figures and images for: TET-catalyzed 5-hydroxymethylcytosine regulates gene expression in differentiating colonocytes and colon cancer
Source: Sci Rep. 2015 Dec 3;5:17568. doi: 10.1038/srep17568 (PMC4668370; doi:10.1038/srep17568)

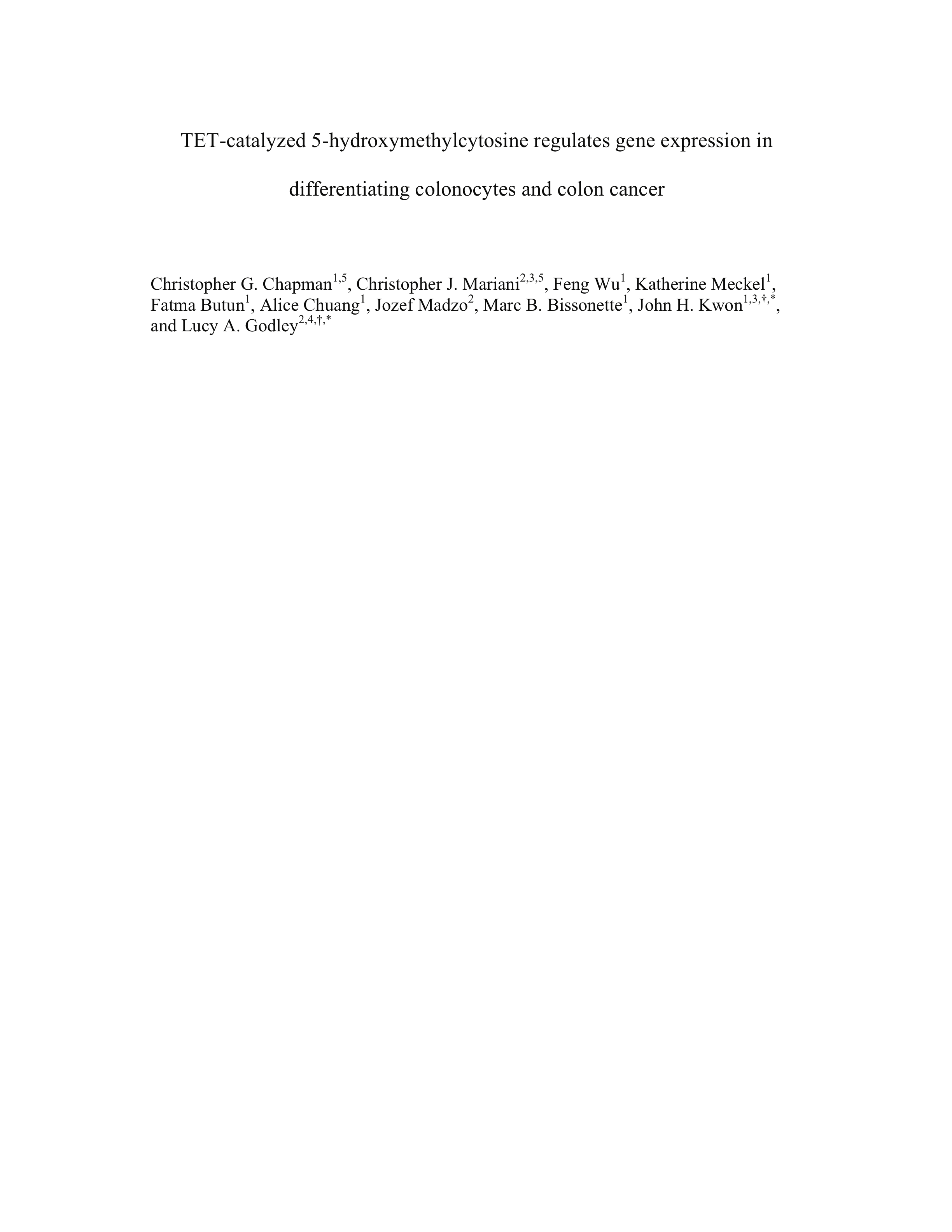


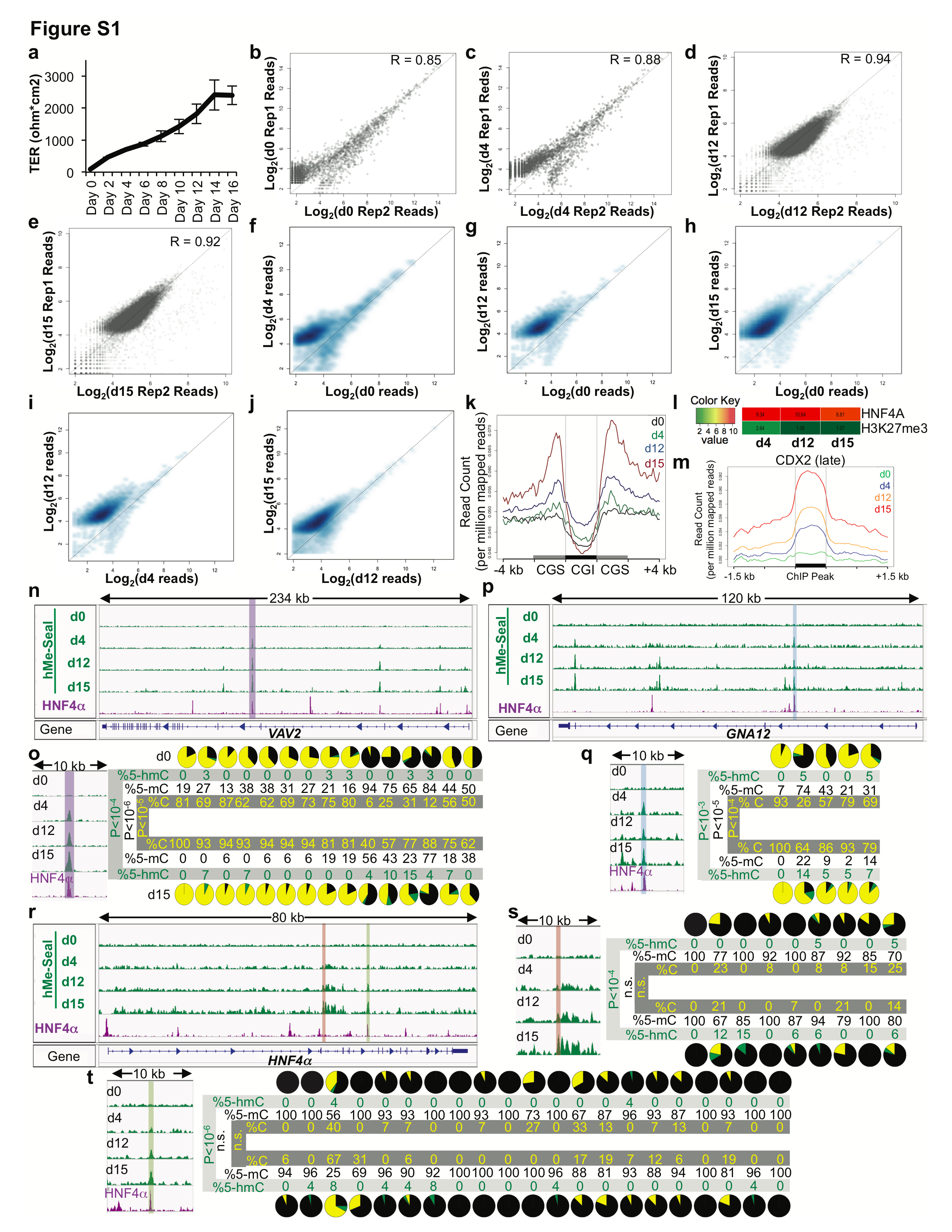


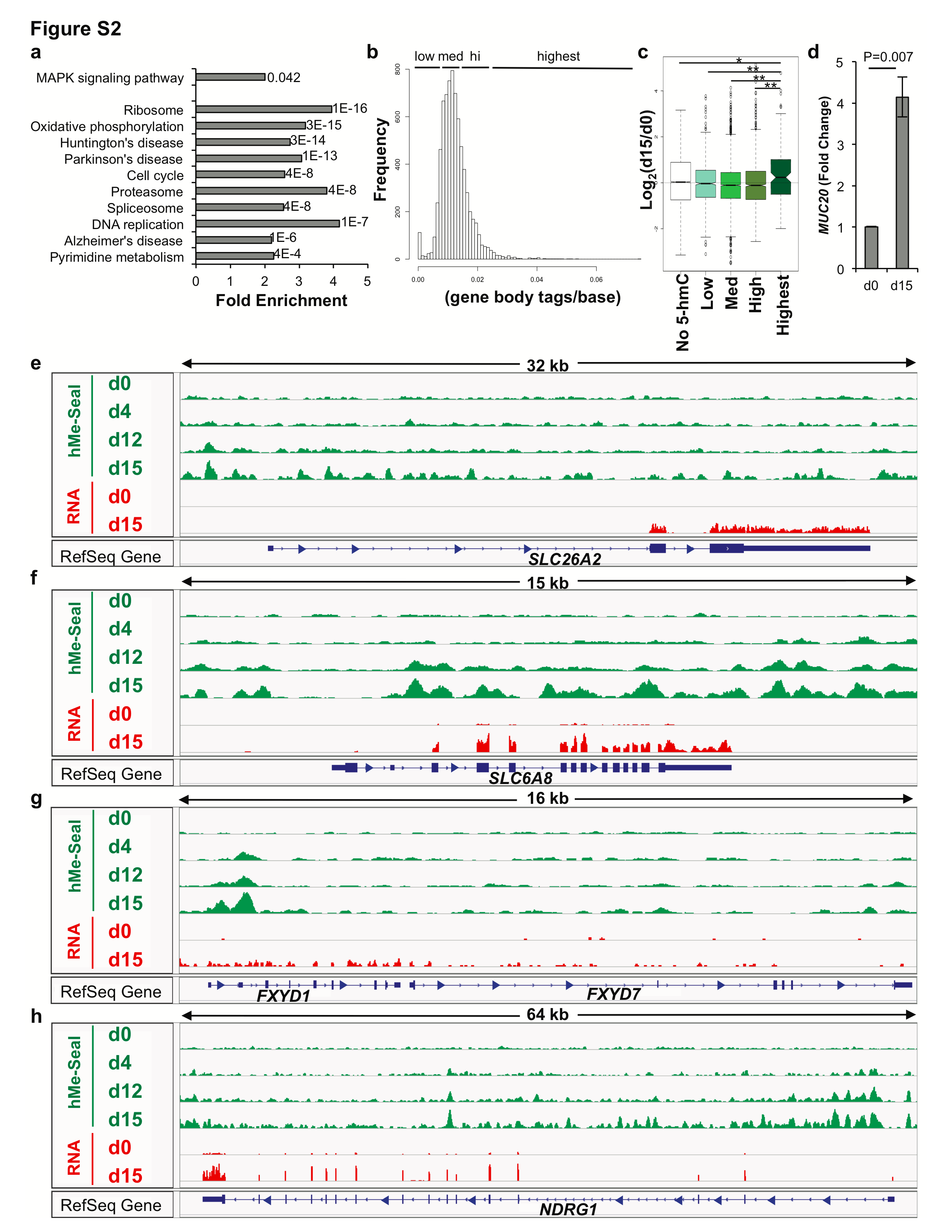


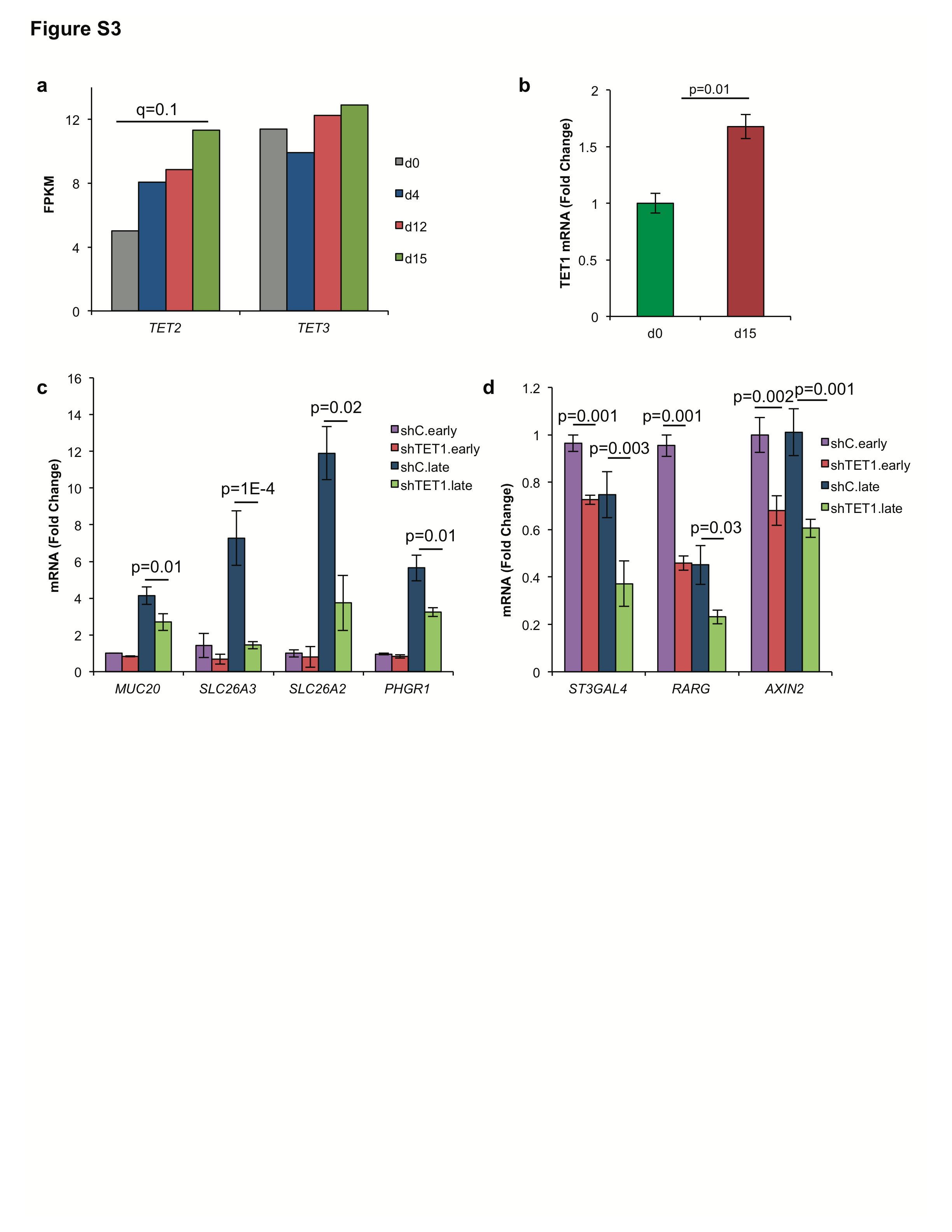


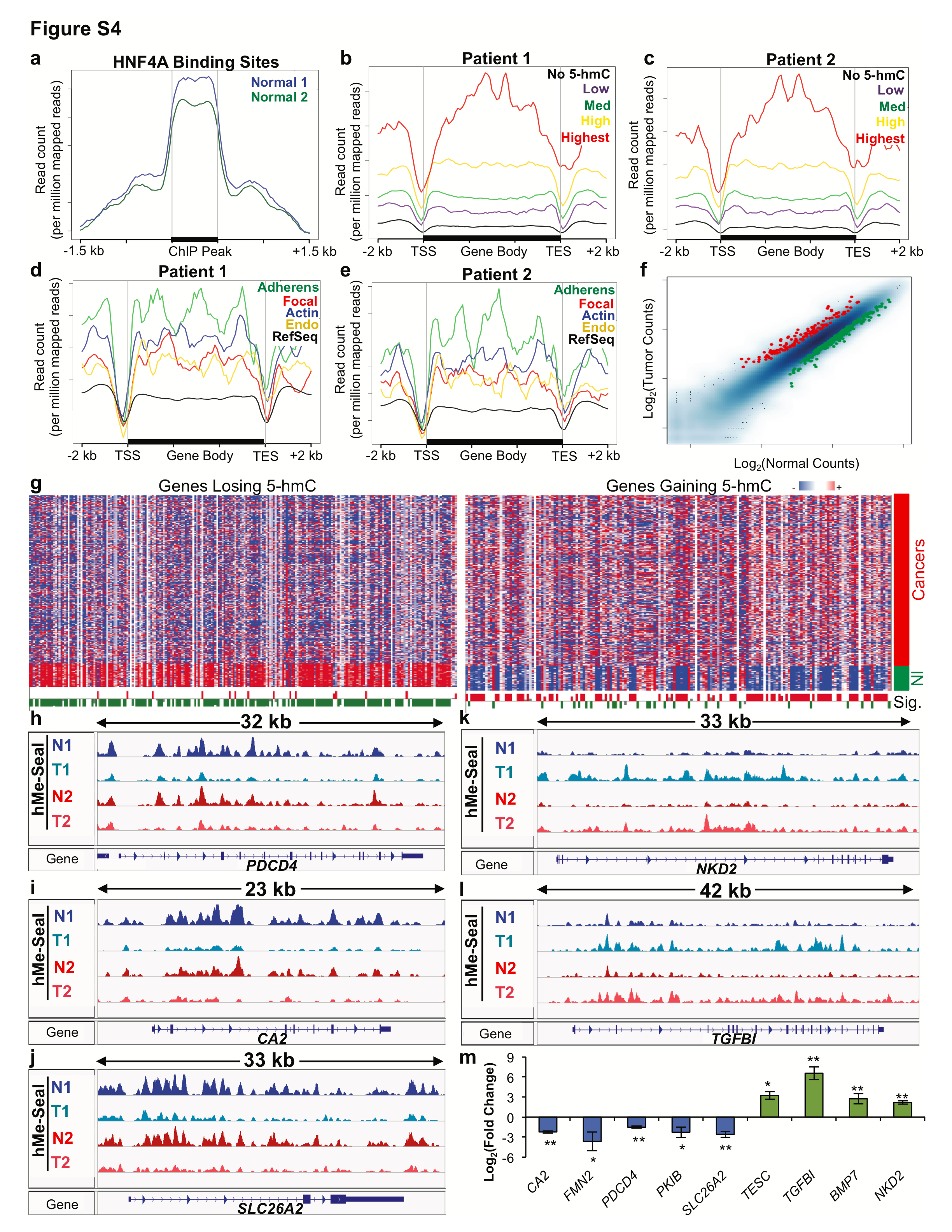


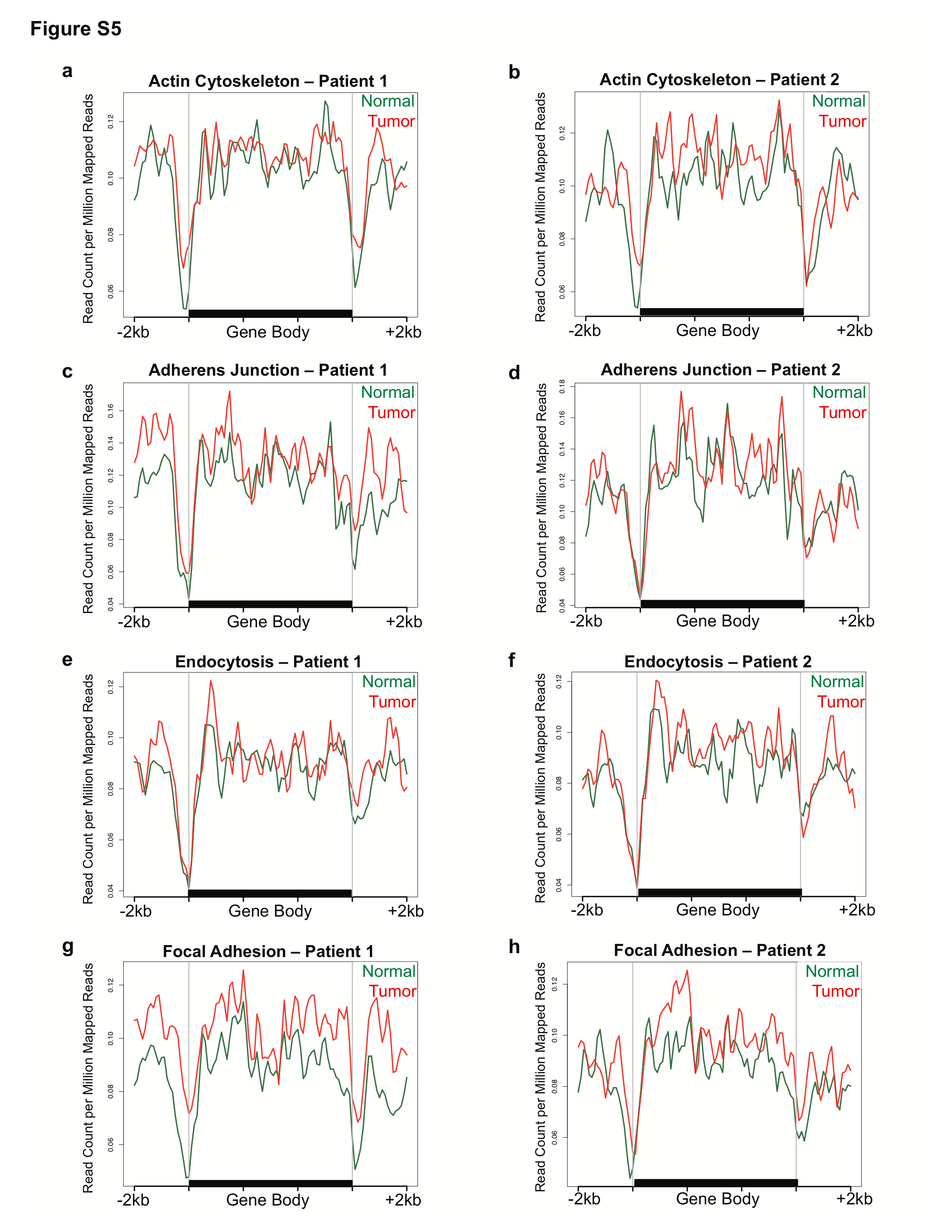


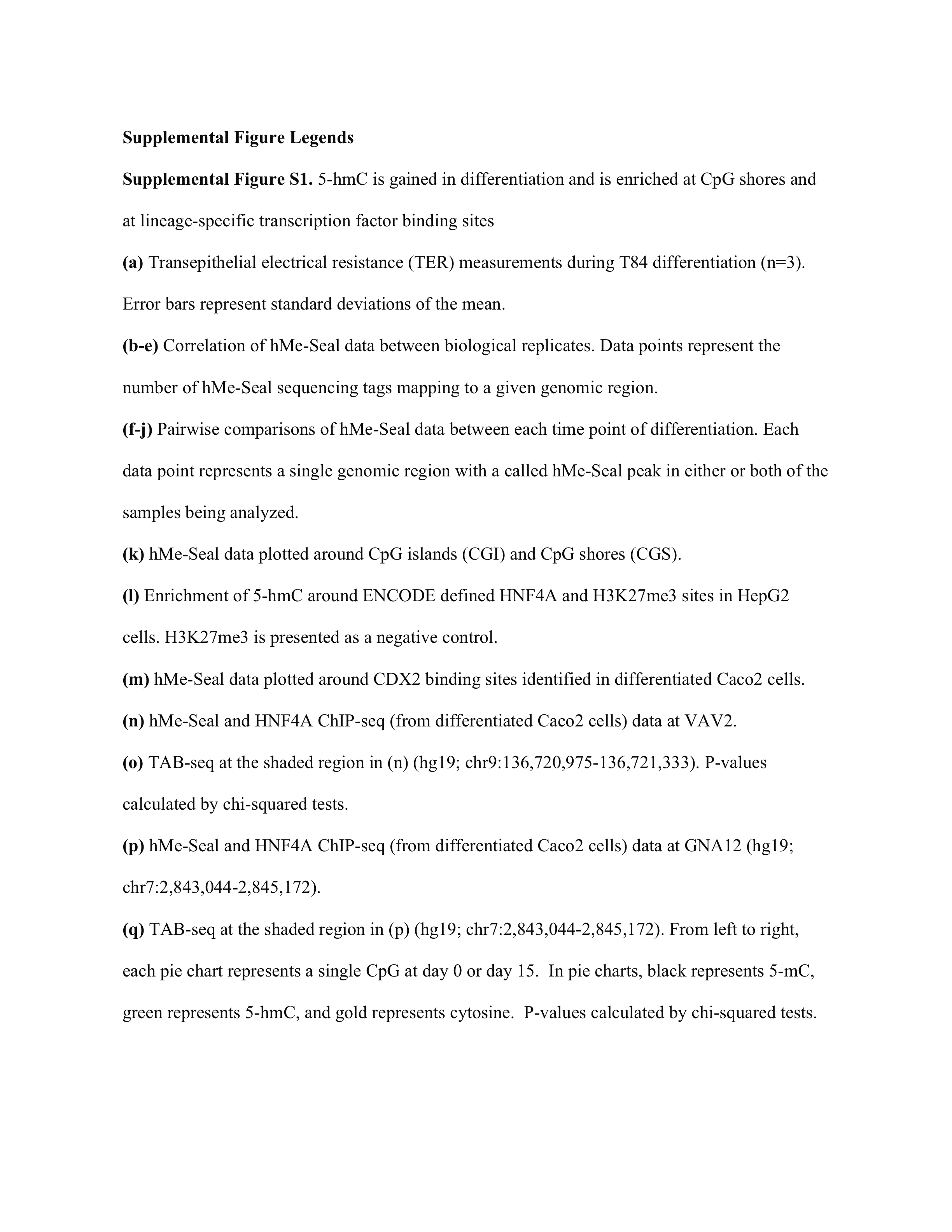


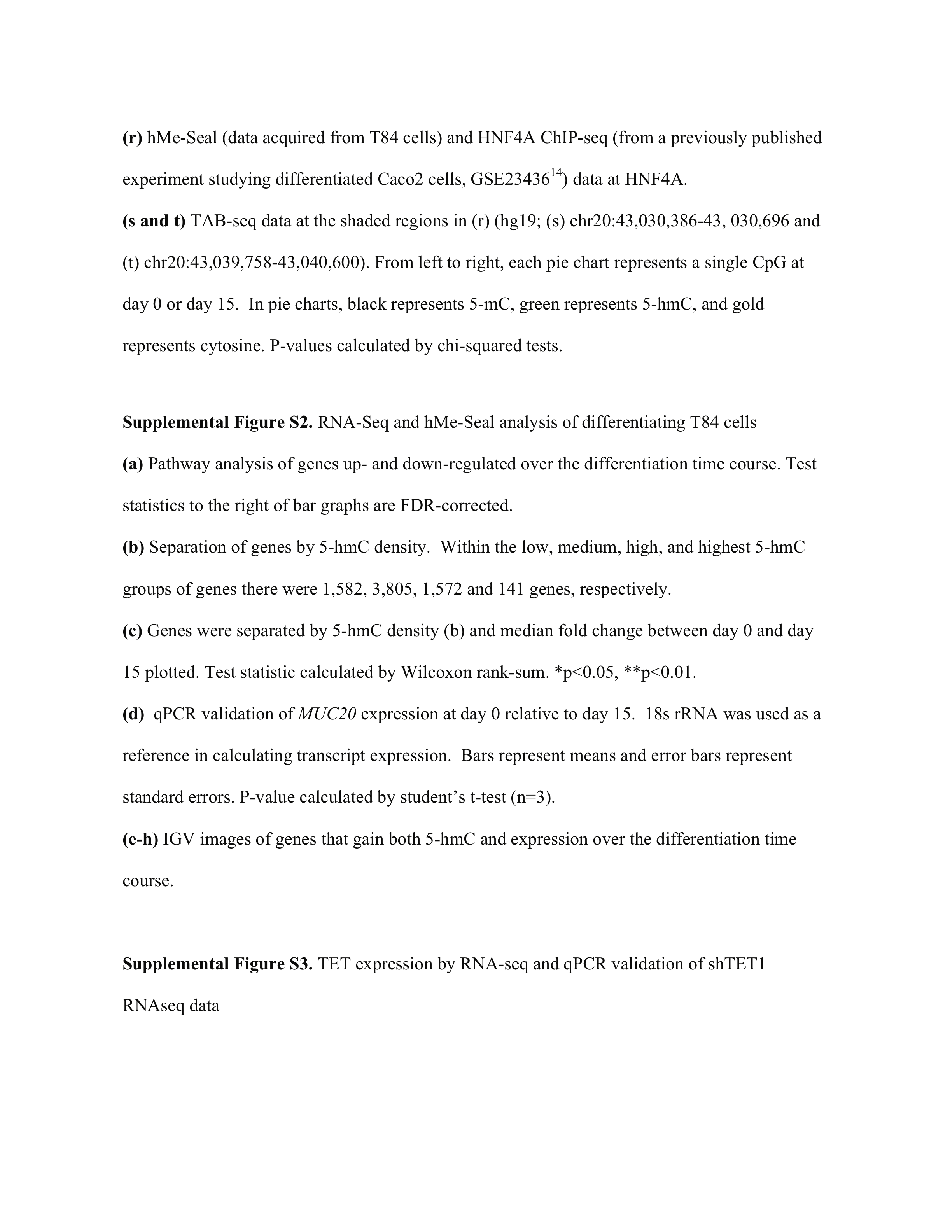


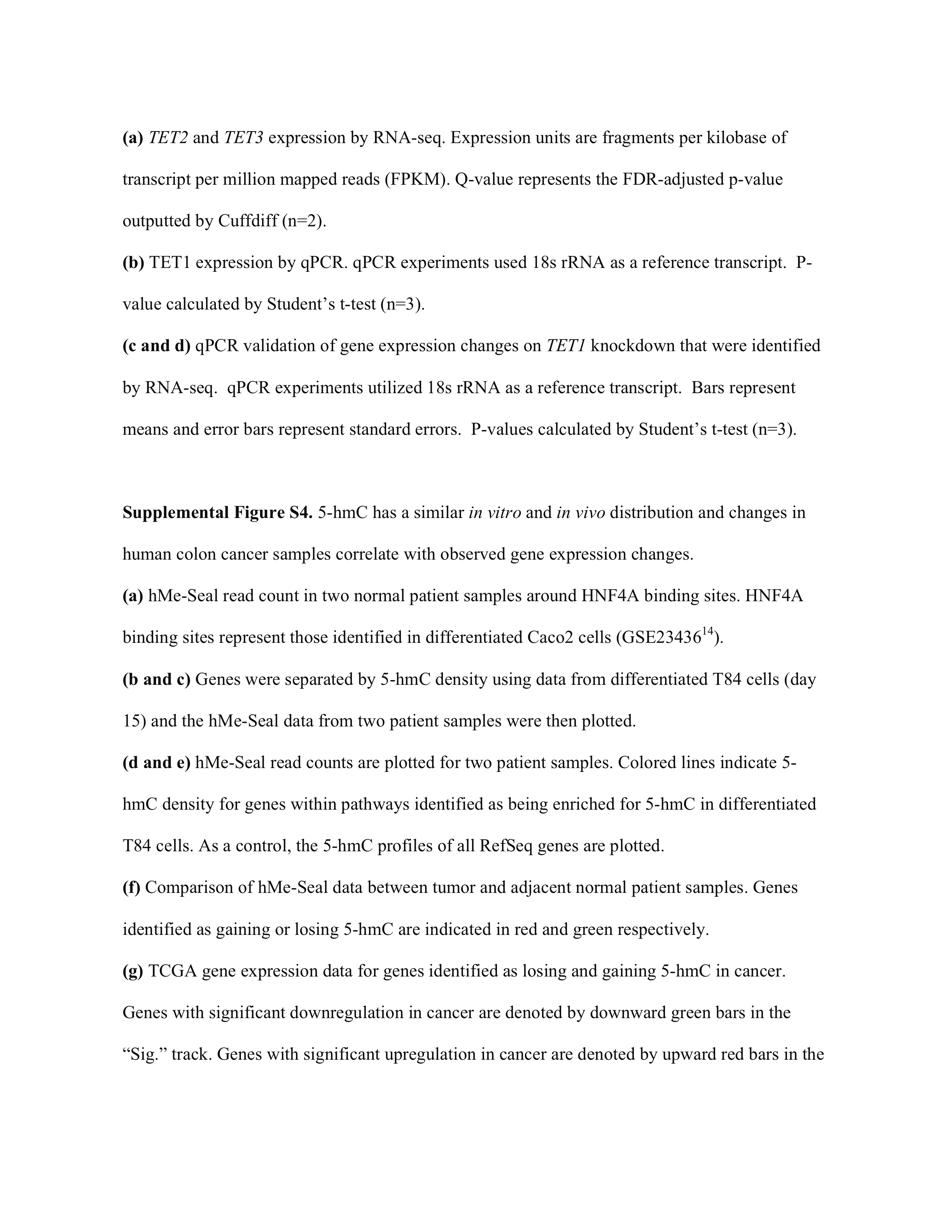


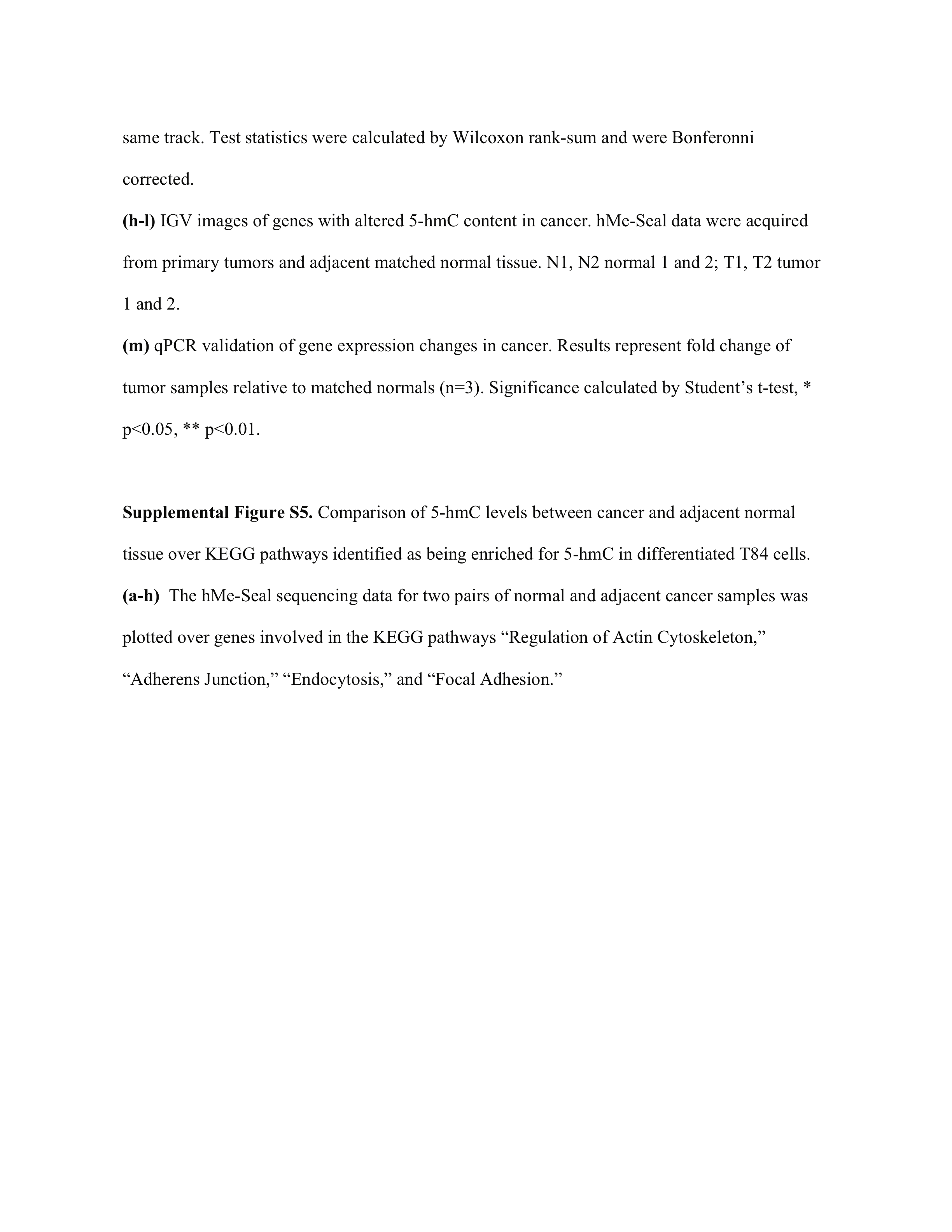


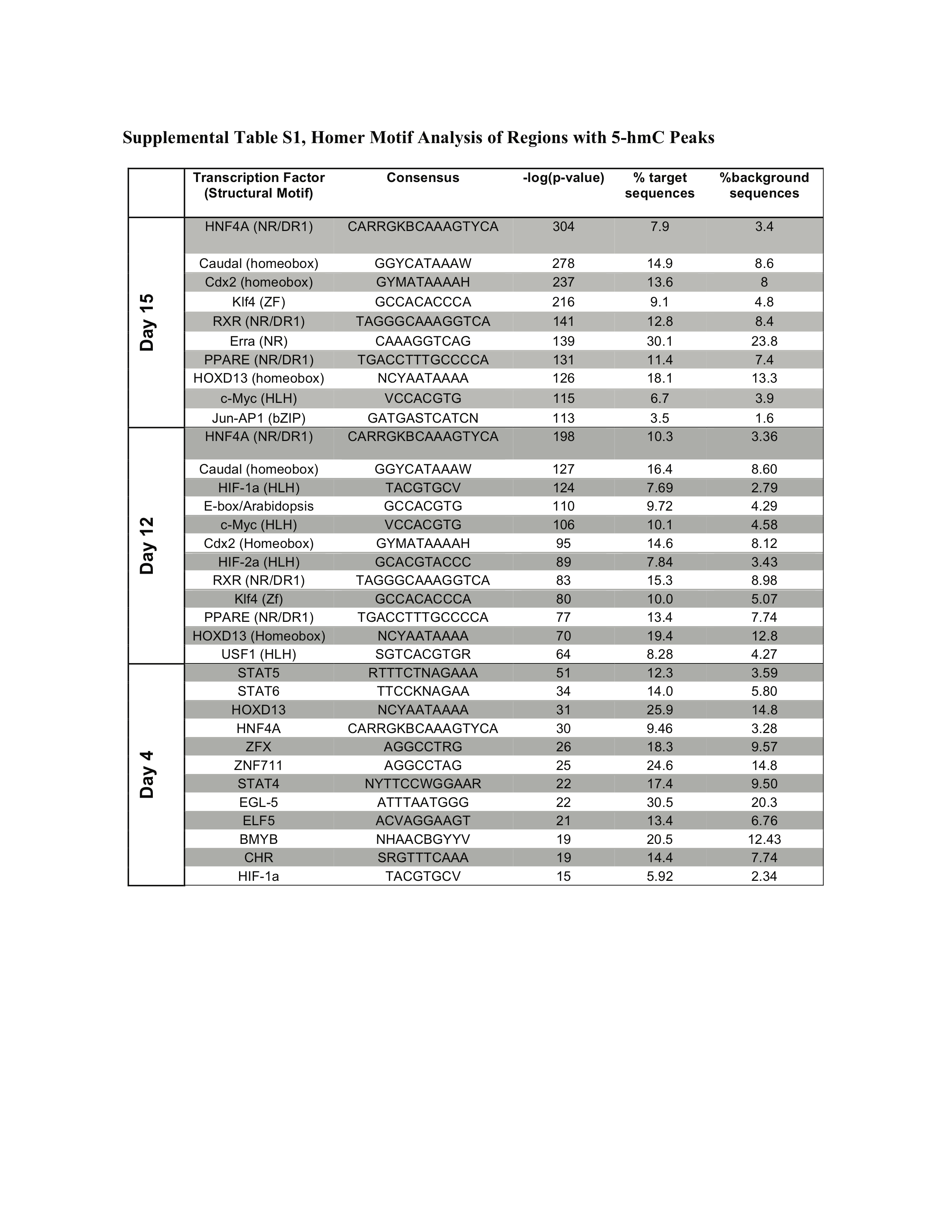

Supplement: Supplementary Data [file srep17568-s1.doc]
